# Fedratinib in patients with myelofibrosis previously treated with ruxolitinib: An updated analysis of the JAKARTA2 study using stringent criteria for ruxolitinib failure

**DOI:** 10.1002/ajh.25777

**Published:** 2020-04-17

**Authors:** Claire N. Harrison, Nicolaas Schaap, Alessandro M. Vannucchi, Jean‐Jacques Kiladjian, Eric Jourdan, Richard T. Silver, Harry C. Schouten, Francesco Passamonti, Sonja Zweegman, Moshe Talpaz, Srdan Verstovsek, Shelonitda Rose, Juan Shen, Tymara Berry, Carrie Brownstein, Ruben A. Mesa

**Affiliations:** ^1^ Guyʼs and St Thomasʼ Hospital London UK; ^2^ Radboud University Nijmegen Medical Centre Nijmegen The Netherlands; ^3^ AOU Careggi University of Florence Florence Italy; ^4^ Hôpital Saint‐Louis Université de Paris; Inserm Paris France; ^5^ Hématologie Clinique Institut de Cancérologie du Gard Nîmes France; ^6^ Weill Cornell Medical College New York New York USA; ^7^ University Hospital Maastricht Maastricht The Netherlands; ^8^ University of Insubria Varese Italy; ^9^ Amsterdam UMC Vrije Universiteit Amsterdam Amsterdam Netherlands; ^10^ University of Michigan Comprehensive Cancer Center Ann Arbor Michigan USA; ^11^ MD Anderson Cancer Center Houston Texas USA; ^12^ Celgene Corporation Summit New Jersey USA; ^13^ University of Texas Health Science Center at San Antonio San Antonio Texas USA

## Abstract

Fedratinib is an oral, selective Janus kinase 2 (JAK2) inhibitor. The phase II JAKARTA2 study assessed fedratinib in patients with intermediate‐ or high‐risk myelofibrosis (MF) who were resistant or intolerant to prior ruxolitinib per investigator assessment. Patients received fedratinib 400 mg/day in 28‐day cycles. The JAKARTA2 outcomes were initially reported using a last‐observation‐carried forward (LOCF) analysis in a “Per Protocol” population. This updated analysis of JAKARTA2 employs intention‐to‐treat analysis principles without LOCF for all treated patients (ITT Population; N = 97), and for a patient subgroup who met more stringent definitions of prior ruxolitinib failure (Stringent Criteria Cohort; n = 79). Median duration of prior ruxolitinib exposure was 10.7 months. The primary endpoint was spleen volume response rate (SVRR; ≥35% spleen volume decrease from baseline to end of cycle 6 [EOC6]). The SVRR was 31% in the ITT Population and 30% in the Stringent Criteria Cohort. Median duration of spleen volume response was not reached. Symptom response rate (≥50% reduction from baseline to EOC6 in total symptom score [TSS] on the modified Myelofibrosis Symptom Assessment Form [MFSAF]) was 27%. Grade 3‐4 anemia and thrombocytopenia rates were 38% and 22%, respectively. Patients with advanced MF substantially pretreated with ruxolitinib attained robust spleen responses and reduced symptom burden with fedratinib.

## 1 INTRODUCTION

Myelofibrosis (MF) is life‐threatening *BCR‐ABL1*‐negative myeloproliferative neoplasm (MPN) characterized by stem cell‐derived clonal myeloproliferation, abnormal cytokine expression, bone marrow fibrosis, splenomegaly, constitutional symptoms, leukemic progression, and poor survival.^1^, ^2^ MF can present de novo (primary MF), or develop secondary to antecedent polycythemia vera (PV) or essential thrombocythemia (ET). MF symptoms, including fatigue, night sweats, pruritus, and splenomegaly‐related symptoms (eg, early satiety and abdominal discomfort or pain) markedly impair quality of life.^3^ For patients with intermediate‐2 or high risk MF per the Dynamic International Prognostic Scoring System Plus (DIPSS‐Plus), estimated median overall survival (OS) is only 2.9 years and 1.3 years, respectively.^4^ Currently, allogeneic stem cell transplantation is the only potentially curative option for MF, but most patients are not eligible for transplant due to comorbidities and general health status.^5^, ^6^, ^7^

In MF, aberrant constitutive activation of the JAK‐signal transducer and activator of transcription (STAT) pathway results in clonal expansion of malignant myeloproliferative cells.^8^ A majority of patients with MF harbor a *JAK2* V617F mutation. Mutations in *JAK2* and in the MPN driver genes, myeloproliferative leukemia virus (*MPL*) and calreticulin (*CALR*), upregulate JAK‐STAT signaling with increased downstream transcription and gene expression. Approximately 10% of patients with MF do not have a *JAK2*, *MPL*, or *CALR* mutation; this is referred to as “triple‐negative” disease.^2^

Until recently, ruxolitinib, a dual JAK1/JAK2 inhibitor, was the only therapy approved for treatment of intermediate‐ and high‐risk MF. Ruxolitinib can improve splenomegaly and symptom scores in patients with primary, post‐PV, or post‐ET MF.^9^, ^10^, ^11^ Many patients treated with ruxolitinib lose response over time, achieve only a suboptimal response, or develop cytopenias during treatment, resulting in therapy discontinuation.^12^ The combined ruxolitinib discontinuation rate in the phase III COMFORT‐I and COMFORT‐II trials was ~50% at 3 years and ~72% at 5 years.^13^, ^14^, ^15^, ^16^ A retrospective review of data from two large United States (US) claims databases suggests ruxolitinib discontinuation rates in clinical practice during early treatment are at least as high as rates in clinical trials.^17^ The prognosis for these patients is generally poor, with median survival ranging from 6 to 28 months, depending on whether a patient is in the chronic phase of MF or has transitioned into the blast phase when ruxolitinib was discontinued.^12^, ^18^, ^19^, ^20^ There is no approved standard of care for patients with MF previously treated with ruxolitinib; thus, there is an important medical need for an effective therapy in this setting.

Fedratinib is an oral kinase inhibitor with activity against wild‐type and mutationally activated *JAK2* and FMS‐like tyrosine kinase 3 (*FLT3*). It was approved by the US Food & Drug Administration (FDA) in August 2019 for treatment of adult patients with intermediate‐2 or high‐risk primary or secondary (post‐PV or post‐ET) MF.^21^ Fedratinib was recently added to the National Comprehensive Care Network guidelines for treatment of MPNs, as an initial treatment option for patients with intermedicate‐2 or high‐risk MF or as second‐line therapy for those who do not respond or lose response to ruxolitinib.^1^ Fedratinib has higher inhibitory activity for JAK2 over family members JAK1, JAK3 and TYK2, and is a more selective inhibitor of JAK2 than ruxolitinib.^22^ Additionally, an in vitro drug screen identified 211 mutations resistant against ruxolitinib that were fully sensitive to fedratinib,^23^ perhaps by a novel mechanism of JAK2 kinase inhibition by fedratinib that prevents emergence of genetic resistance, making it a therapeutic option for patients who are resistant to ruxolitinib therapy. Fedratinib also has a longer effective half‐life than ruxolitinib (~41 hours vs 3 hours, respectively), which allows more persistent JAK2 inhibition and makes it suitable for once‐daily dosing.^24^, ^25^

The international, single‐arm phase II JAKARTA2 trial evaluated the efficacy and safety of fedratinib in patients with intermediate‐ or high‐risk primary MF, post‐PV MF, or post‐ET MF, who were previously treated with ruxolitinib. The fedratinib clinical development program was placed on clinical hold in November 2013 by the US FDA following reports of suspected Wernickeʼs encephalopathy (WE), a rare but serious neurological condition. As a result, the JAKARTA2 trial was terminated; all patients were required to discontinue fedratinib treatment and the study was substantially truncated.

Note, JAKARTA2 enrolled patients who were resistant or intolerant to prior ruxolitinib therapy based on investigator assessment. The primary endpoint was the spleen volume response rate (SVRR); that is, the proportion of patients who achieved a ≥35% reduction from baseline spleen volume at the end of cycle 6 (EOC6). Based on the prospective Statistical Analysis Plan, the primary efficacy analysis of JAKARTA2 was performed in the Per Protocol population, which comprised patients with spleen volume assessments at baseline and at least one post‐baseline time point. The original analysis utilized a last‐observation‐carried‐forward (LOCF) analysis method, which allowed for the last spleen volume assessment to be “carried forward” for patients missing EOC6 assessments.^26^ At EOC6, the SVRR (using LOCF method) in the Per Protocol population was 55% (95% confidence interval [CI] 44, 66).^26^

The objectives of this updated analysis are to confirm the efficacy of fedratinib in the JAKARTA2 study by employing intention‐to‐treat (ITT) analysis principles for all treated patients. There is no imputation made for missing data, and to demonstrate efficacy outcomes in a subgroup of JAKARTA2 patients who met new, more stringent criteria for relapsed, refractory, or intolerant to ruxolitinib than were used in the original analysis. Additionally, a sensitivity analysis assessed fedratinib efficacy in patients who met the more stringent criteria for ruxolitinib failure, and for whom the primary endpoint would have been least affected by early termination of the study.

## 2 METHODS

The phase II, international, multicenter, open‐label, single‐arm JAKARTA2 study (ClinicalTrials.gov NCT01523171) was conducted at 40 sites in 10 countries. The study protocol was approved by relevant independent ethics committees or institutional review boards at each site. All patients provided written, informed consent before study participation. Detailed study design and inclusion/exclusion criteria have been described.^26^ Briefly, eligible patients were aged ≥18 years, with primary, post‐PV, or post‐ET MF; intermediate‐1 (with symptoms), intermediate‐2, or high risk disease; palpable splenomegaly (≥5 cm below the left costal margin); Eastern Cooperative Oncology Group (ECOG) performance status scores ≤2; platelet counts ≥50 × 10^9^/L, and were considered by their treating investigator to be resistant or intolerant to ruxolitinib (Table S1).

Patients received oral fedratinib 400 mg once‐daily in repeated 28‐day treatment cycles. Dose escalation was permitted up to 600 mg/day if there was <50% reduction in spleen size by palpation at the end of cycles 2 and 4, and the fedratinib dose could be reduced, interrupted, or discontinued in cases of toxicity.

This updated analysis assesses three patient populations (Table S1): the *ITT Population* includes all patients who enrolled in JAKARTA2; the *Stringent Criteria Cohort* comprises a subset of patients who met the new, more stringent criteria for relapsed or refractory to ruxolitinib (based on spleen volume or size assessments), or intolerant to ruxolitinib, than used in the original analysis.^26^ The *Sensitivity Analysis Cohort* includes the subgroup of patients within the Stringent Criteria Cohort who were least affected by early study termination, that is, those who reached fedratinib treatment cycle six, or discontinued fedratinib before cycle six for reasons other than “*study terminated by the sponsor*”. These criteria were presented to and accepted by MF experts from the United States and European Union at an advisory board meeting with the study sponsor. The sponsors also reviewed the proposed criteria with relevant health authorities.

### 2.1 Efficacy endpoints

The primary endpoint was SVRR, defined as the proportion of patients who achieved a ≥ 35% reduction in spleen volume from baseline to EOC6. Spleen volume assessments were to be performed at baseline, at the end of cycles three and six, and every six cycles thereafter. Blinded review of MRI/CT scans was performed by an independent central imaging laboratory. This updated analysis employed ITT analysis principles; thus, missing spleen volume data were not imputed (no LOCF) for the primary endpoint, and patients missing assessments at EOC6 were considered nonresponders. No formal statistical adjustments were made for possible covariate effects or for multiple comparisons.

SVRR at EOC6 was also evaluated in patient subgroups defined by baseline platelet count (50 to <100 × 10^9^/L or ≥100 × 10^9^/L), baseline hemoglobin level (<10 or ≥10 g/dL), number of prior therapies (≤2 or >2), age (≤65 or >65 years), JAK2 mutation status (mutant or wild‐type), and outcome of prior ruxolitinib treatment per new stringent criteria (relapsed, refractory, or intolerant).

A key secondary endpoint was symptom response rate, defined as the proportion of patients with a ≥50% decrease in total symptom score (TSS) from baseline to EOC6. Symptom scores were subjectively evaluated using the modified Myelofibrosis Symptom Assessment Form (MFSAF^27^) e‐diary, which assesses the severity of six key MF‐associated symptoms (night sweats, pruritis, abdominal discomfort, early satiety, pain under ribs on left side, and bone or muscle pain), each assigned a score from 0 (absent) to 10 (worst imaginable). TSS is the sum of individual symptom scores. The MFSAF was to be completed beginning 7 days before cycle 1‐day 1, and then 7 days before day 1 of each subsequent treatment cycle for six treatment cycles, and at EOC6. The MFSAF Analysis Population included patients with evaluable TSS data available at baseline and at least 1 post‐baseline assessment. Confidence intervals for spleen volume and symptom response rates were calculated using the Clopper‐Pearson Exact method.

Additional secondary endpoints included the duration of spleen volume response, calculated from the date of first response to the date of disease progression (≥25% spleen volume increase from baseline) or death, whichever came first. Duration of spleen response was estimated by Kaplan‐Meier (KM) analysis among patients who responded at any time on‐study. In the absence of disease progression or death before the analysis cut‐off date (May 7, 2014), duration of response was censored at the date of the last valid assessment before data cutoff. Also assessed were median percent change in spleen volume from baseline to EOC6, proportion of patients with ≥50% reductions in spleen size by palpation at EOC6, and proportion of patients with ≥35% reduction from baseline in spleen volume at EOC3.

### 2.2 Safety

The safety and tolerability of fedratinib were evaluated based on the incidence of treatment‐emergent adverse events (TEAEs), classified according to the Medical Dictionary for Regulatory Activities (MedDRA) version 20.1, and hematologic and biochemical laboratory values. The TEAEs (preferred terms unless otherwise noted) were graded according to National Cancer Institute Common Terminology Criteria for Adverse Events (NCI‐CTCAE) version 4.03. A TEAE was defined as any AE that developed, worsened, or became serious between first dose of fedratinib to 30 days after the last dose. Although transfusions were allowed, concomitant use of anti‐anemic preparations (eg, erythropoietin and darbepoetin) was not permitted on‐study.

## 3 RESULTS

### 3.1 Patients

In all, 97 patients were enrolled and treated in JAKARTA2 between 30 April 2012, and 7 May 2014, and comprise the ITT Population (Figure S1). The majority of patients (n = 63; 65%) discontinued treatment due to study termination following the fedratinib clinical hold. Other common reasons for treatment discontinuation were adverse events (19%) and disease progression (6%).

Based on new, more stringent criteria for ruxolitinib failure, 79/97 patients (81%) were refractory (n = 47; 48%), relapsed (n = 18; 19%), or intolerant (n = 14; 14%) to prior ruxolitinib therapy and comprised the Stringent Criteria Cohort. The remaining 18 patients were excluded from the Stringent Criteria Cohort because they had an adequate response to ruxolitinib (n = 3), were missing ruxolitinib response data (n = 8), or did not receive ≥3 months of ruxolitinib treatment (n = 7). The Sensitivity Analysis Cohort included 66 patients within the Stringent Criteria Cohort who had the opportunity to receive six cycles of fedratinib therapy or discontinued treatment prior to cycle six for reasons other than study termination.

The median age of all patients was 67 years (range 38‐83). At entry, patients generally had poor prognostic disease characteristics (Table 1). Median baseline spleen volume was 2894 mL (~14 times that reported in the healthy population^28^) and 93 patients (96%) reported experiencing one or more MFSAF symptom at baseline. The majority (79%) of patients had received ≥2 prior MF‐directed therapies, and 13% had received ≥4 MF‐directed therapies before study entry. One‐third of all patients had baseline platelet counts of 50 to <100 × 10^9^/L. Over one‐half (53%) of patients had baseline hemoglobin levels <10 g/dL and 14% were RBC transfusion‐dependent.^29^ There were no overt differences in baseline characteristics between the ITT Population and the Stringent Criteria or Sensitivity Analysis cohorts (Table 1).

**TABLE 1:** Baseline characteristics

Parameter	ITT population (N = 97)	Stringent criteria cohort (n = 79)	Sensitivity analysis cohort (n = 66)
Age, years, median (range)	67 (38‐83)	66 (38‐83)	66 (38‐83)
Disease type, n (%)			
Primary MF	53 (55)	47 (60)	38 (58)
Post‐PV MF	25 (26)	18 (23)	17 (26)
Post‐ET MF	19 (20)	14 (18)	11 (17)
Risk status, n (%)			
Intermediate‐1 with symptoms	16 (17)	11 (14)	6 (9)
Intermediate‐2	47 (49)	41 (52)	35 (53)
High	34 (35)	27 (34)	25 (38)
Years since MF diagnosis, median (range)	4.1 (0.3‐24.5)	5.4 (0.4‐24.5)	5.6 (0.4–24.5)
Prior ruxolitinib exposure, months, median (range)	10.7 (0.1–62.4)	11.5 (1.0–62.4)	11.5 (1.0–62.4)
RBC transfusion dependence, n (%)	14 (14)	13 (17)	12 (18)
MFSAF symptoms,^a^ n (%)			
Yes	93 (96)	76 (96)	64 (97)
No	4 (4)	3 (4)	2 (3)
*JAK2* mutational profile, n (%)			
Mutant	61 (63)	48 (61)	41 (62)
Wild‐type	29 (30)	25 (32)	20 (30)
Missing	7 (7)	6 (8)	5 (8)
Platelet count, n (%)			
50 to <100 × 10^9^/L	33 (34)	28 (35)	26 (39)
≥100 × 10^9^/L	64 (66)	51 (65)	40 (61)
Hemoglobin level, n (%)			
<10 g/dL	51 (53)	46 (58)	40 (61)
≥10 g/dL	46 (47)	33 (42)	26 (39)
Spleen volume, mL, median (range)	2894 (737‐7815)	2946 (737–7815)	2998 (784‐7815)
Spleen size, cm, median (range)	18 (5‐36)	18 (5‐36)	18 (5‐36)

Abbreviations: ET, essential thrombocythemia; ITT, intention‐to‐treat; JAK2, Janus kinase 2; MF, myelofibrosis; PV, polycythemia vera; RBC, red blood cell.

a Night sweats, itching, abdominal discomfort, abdominal pain, early satiety, or bone pain.

In the ITT Population, the median duration of prior ruxolitinib treatment was 10.7 months (range 0.1‐62.4). Most patients (71%) had received ruxolitinib at initial daily doses of 30‐40 mg, with median cumulative ruxolitinib exposure in the ITT Population of 9540 mg (range 80‐50 480 mg). Median duration of prior ruxolitinib exposure in the both Stringent Criteria and Sensitivity Analysis subgroups was 11.5 months (range 1.0‐62.4 months).

The median number of fedratinib cycles received in the ITT Population at the time of the clinical hold was six (range 1‐20 cycles) and median actual fedratinib dose intensity was 2000 mg/week (1403‐3884 mg). Median number of fedratinib treatment cycles in the Stringent Criteria and Sensitivity Analysis cohorts was seven (range 1‐20 cycles).

### 3.2 Efficacy

#### 3.2.1 Spleen response

In the ITT Population, SVRR was 31% (95%CI 22%, 41%). Response rates in the Stringent Criteria and Sensitivity Analysis cohorts supported the robustness of efficacy findings in the ITT Population: SVRR was 30% (95%CI 21%, 42%) in the Stringent Criteria Cohort and 36% (25%, 49%) in the Sensitivity Analysis Cohort (Table 2). In subgroup analyses, SVRRs were not significantly influenced by reason for prior ruxolitinib failure (relapsed/refractory or intolerant), number of prior anti‐cancer therapies, platelet count, hemoglobin level, patient age, or *JAK2* mutational status (Table 2). In patients with platelet counts of 50 to <100 × 10^9^/L at study entry, SVRRs in the ITT Population, Stringent Criteria Cohort, and Sensitivity Analysis Cohort were 36%, 39%, and 42%, respectively; and SVRRs in patients with hemoglobin levels <10 g/dL at baseline were 28%, 26%, and 30% (Table 2).

**TABLE 2:** Subgroup analyses: Spleen volume response rates (SVRR) at end of cycle six, overall and in subgroups defined by patient characteristics at baseline

	ITT population (N = 97)		Stringent criteria cohort (n = 79)		Sensitivity analysis cohort (n = 66)	
**SVRR at EOC6 (overall), n (%)**	30 (31%)		24 (30%)		24 (36%)	
**[95% CI]**	[22, 41]		[21, 42]		[25, 49]	

Prior ruxolitinib outcome	Resistant^a^	Intolerant^a^	Relapsed/refractory^b^	Intolerant^b^	Relapsed/refractory^b^	Intolerant^b^
	n = 64	n = 32	n = 65	n = 14	n = 56	n = 10
SVRR, n (%)	21 (33%)	9 (28%)	20 (31%)	4 (29%)	20 (36%)	4 (40%)
[95% CI]	[22, 46]	[14, 47]	[20, 43]	[8, 58]	[23, 50]	[12, 74]

Number of prior therapies	≤2	>2	≤2	>2	≤2	>2
	n = 67	n = 30	n = 58	n = 21	n = 49	n = 17
SVRR, n (%)	23 (34%)	7 (23%)	19 (33%)	5 (24%)	19 (39%)	5 (29%)
[95% CI]	[23, 47]	[10, 42]	[21, 46]	[8, 47]	[25, 54]	[10, 56]

Platelet count (10^9^/L)	50 to <100	≥100	50 to <100	≥100	50 to <100	≥100
	n = 33	n = 64	n = 28	n = 51	n = 26	n = 40
SVRR, n (%)	12 (36%)	18 (28%)	11 (39%)	13 (26%)	11 (42%)	13 (33%)
[95% CI]	[20, 55]	[18, 41]	[22, 59]	[14, 40]	[23, 63]	[19, 49]

Hemoglobin concentration (g/dL)	<10	≥10	<10	≥10	<10	≥10
	n = 51	n = 46	n = 46	n = 33	n = 40	n = 26
SVRR, n (%)	14 (28%)	16 (35%)	12 (26%)	12 (36%)	12 (30%)	12 (46%)
[95% CI]	[16, 42]	[21, 50]	[14, 41]	[20, 55]	[17, 47]	[27, 67]

Patient age	≤65	>65	≤65	>65	≤65	>65
	n = 41	n = 56	n = 36	n = 43	n = 32	n = 34
SVRR, n (%)	14 (34%)	16 (29%)	12 (33%)	12 (28%)	12 (38%)	12 (35%)
[95% CI]	[20, 51]	[17, 42]	[19, 51]	[15, 44]	[21, 56]	[20, 54]

*JAK2* mutation status	Mutant	Wild‐type	Mutant	Wild‐type	Mutant	Wild‐type
	n = 61	n = 29	n = 48	n = 25	n = 41	n = 20
SVRR, n (%)	23 (38%)	5 (18%)	17 (35%)	5 (20%)	17 (42%)	5 (25%)
[95% CI]	[26, 51]	[6, 36]	[22, 51]	[7, 41]	[26, 58]	[9, 49]

Abbreviations: 95% CI, 95% confidence interval; EOC, end of cycle; ITT, intention‐to‐treat; JAK2, Janus kinase 2; SVRR, spleen volume response rate.

a Per enrolling investigator. One patient was classified as “Other: lack of efficacy.”

b Relapsed/refractory or intolerant per updated stringent criteria (see Table S1).

The duration of spleen response was subject to extensive censoring due to early study termination; follow‐up ranged from 0 to 13.4 months. Among patients who achieved a spleen response at any time on‐study (n = 47), the estimated median duration of response was not reached (NR; 95%CI 7.2 months, NR) (Figure S2), and only two responders (4%) experienced disease progression or died by the time of study termination. Only 25% of the 47 responders in the ITT Population had a duration of response of less than 9.4 months. Median spleen volume response duration was also NR (95%CI 7.2 months, NR) in both the Stringent Criteria Cohort (n = 41 responders) and the Sensitivity Analysis Cohort (n = 34 responders).

**FIGURE 1:**
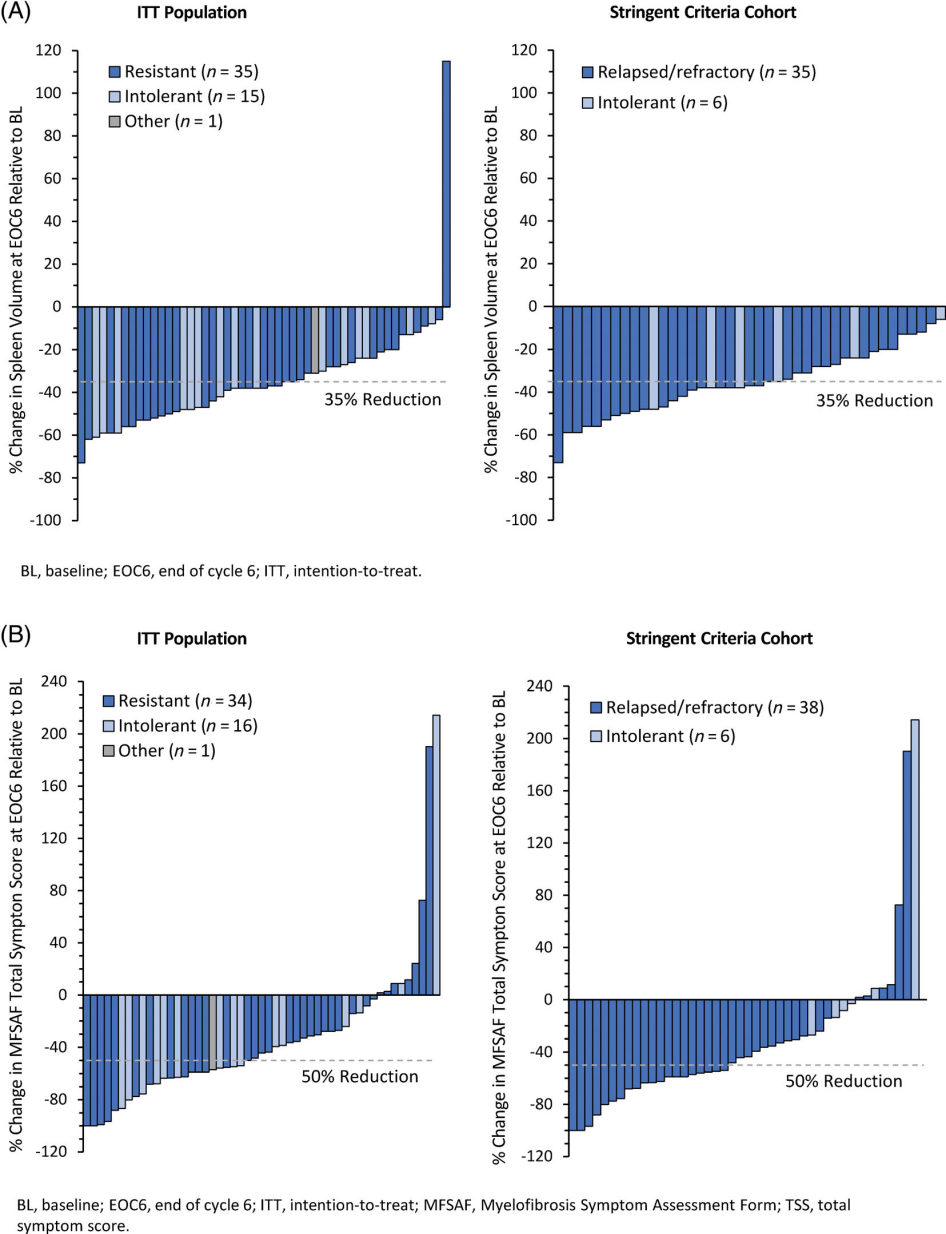
Waterfall plot of individual changes from baseline in spleen volume, A and symptom score, B, in patients with assessments at baseline and end of cycle 6 (EOC6)

At EOC6, median percent change in spleen volume from baseline was −38% (range –73% to +115%) in the ITT Population and –37% (−73% to −6%) in both the Stringent Criteria and Sensitivity Analysis cohorts. Among 51 patients in the ITT Population who had spleen volume assessments at both baseline and at EOC6, all but one (98%) achieved some degree of reduction in spleen volume with fedratinib, and all patients in the Stringent Criteria Cohort with assessments at both timepoints (by definition, the Sensitivity Analysis Cohort) had spleen volume reductions (Figure 1A). Reductions of ≥50% in spleen size by palpation at EOC6 occurred in 31% of patients (n = 30) in the ITT Population, 30% (n = 24) in the Stringent Criteria Cohort, and 36% (n = 24) in the Sensitivity Analysis Cohort.

Proportions of patients with ≥35% reductions from baseline in spleen volume at EOC3 in the ITT Population, Stringent Criteria Cohort, and Sensitivity Analysis Cohort were 40% (95%CI 30, 51), 43% (32, 55), and 41% (29, 54), respectively.

#### 3.2.2 Symptom response

The symptom response rate in the MFSAF Analysis Population (n = 90) was 27% (95%CI 18, 37). Among patients with evaluable TSS data at baseline and EOC6, 82% reported some decrease in symptom severity with fedratinib (Figure 1B). Clinically meaningful improvements in symptom scores were observed across all individual symptoms (Table S2). Symptom response rates in the Stringent Criteria Cohort (n = 74) and in the Sensitivity Analysis Cohort (n = 62) supported results for the ITT Population; at EOC6, symptom response rates were 27% (95%CI 17, 39) and 32% (21, 45), respectively.

Baseline platelet count did not appear to influence symptom score reductions with fedratinib: symptom response rate at EOC6 was 39% (95%CI 22%, 58%) in the subgroup of patients with baseline platelet counts of 50 to <100 × 10^9^/L, and 20% (11%, 33%) in patients with platelet counts ≥100 × 10^9^/L.

### 3.3 Safety

All 97 patients experienced at least one TEAE. The most commonly reported non‐hematologic TEAEs (any grade) were diarrhea (62%), nausea (56%), vomiting (41%), constipation (21%), pruritus (18%), and fatigue (16%). The most common hematological TEAEs were anemia (49%) and thrombocytopenia (27%) (Table 3). Grade 3 or grade 4 TEAEs were reported for 63% of patients; rates of grade 3‐4 anemia and thrombocytopenia were 38% and 22%, respectively. In laboratory assessments, the most commonly reported abnormalities were anemia (99%), creatinine increase (74%), and thrombocytopenia (70%) (Table S3).

**TABLE 3:** Treatment‐emergent adverse events (TEAEs) reported in >10% of patients in the ITT population

Preferred term	ITT population (N = 97)		Stringent criteria cohort (n = 79)		Sensitivity analysis cohort (n = 66)	
	Any Grade n (%)	Grade 3–4 n (%)	Any Grade n (%)	Grade 3–4 n (%)	Any Grade n (%)	Grade 3–4 n (%)
Diarrhea	60 (62)	4 (4)	51 (65)	3 (4)	44 (67)	3 (5)
Nausea	54 (56)	0	42 (53)	0	35 (53)	0
Anemia	47 (49)	37 (38)	44 (56)	35 (44)	39 (59)	31 (47)
Thrombocytopenia	26 (27)	21 (22)	21 (27)	16 (20)	20 (30)	15 (23)
Vomiting	40 (41)	0	35 (44)	0	31 (47)	0
Constipation	20 (21)	1 (1)	17 (22)	0	15 (23)	0
Pruritus	17 (18)	0	14 (18)	0	12 (18)	0
Fatigue	15 (16)	2 (2)	11 (14)	1 (1)	10 (15)	1 (2)
Cough	13 (13)	0	12 (15)	0	9 (14)	0
Headache	13 (13)	1 (1)	10 (13)	1 (1)	9 (14)	1 (2)
Urinary tract infection	12 (12)	0	11 (14)	0	11 (17)	0
Abdominal pain	12 (12)	2 (2)	9 (11)	1 (1)	8 (12)	1 (2)
Dyspnea	12 (12)	1 (1)	9 (11)	1 (1)	8 (12)	1 (2)
Asthenia	11 (11)	1(1)	10 (13)	1 (1)	7 (11)	1 (2)
Dizziness	11 (11)	0	9 (11)	0	7 (11)	0
Pyrexia	11 (11)	1 (1)	7 (9)	0	7 (11)	0

*Note:* TEAEs were classified according to the Medical Dictionary for Regulatory Activities (MedDRA) version 20.1, and graded according to National Cancer Institute Common Terminology Criteria for Adverse Events (NCI‐CTCAE) version 4.03.

Abbreviations: NR, not reported; TEAE, treatment‐emergent adverse event.

Serious TEAEs were reported for 33 patients (34%), the most common being pneumonia (4%) and pleural effusion (3%). Eleven patients experienced a serious event that was considered treatment‐related; pneumonia was the only treatment‐related serious TEAE reported for more than one patient (n = 2).

Seven patients (7%) experienced a TEAE that led to death during the treatment period or the 30‐day follow‐up period. The cause of death was determined to be disease progression in four cases, and the other three cases were due to TEAEs (pneumonia, cardiorespiratory arrest, and shock) that were not considered to be related to study treatment.

Proportions of patients with treatment interruptions of ≥7 days or any fedratinib dose‐reduction were 26% and 39%, respectively. The most common reasons for interruptions or dose‐reductions were nausea (8%), anemia (8%), diarrhea (7%), and thrombocytopenia (6%). Nineteen patients (20%) permanently discontinued fedratinib due to a TEAE (regardless of causality); diarrhea and thrombocytopenia (n = 2 each) were the only TEAEs leading to discontinuation in >1 patient. Treatment‐related TEAEs led to permanent treatment discontinuation for 10 patients (10%), eight of whom had a grade 3 or grade 4 treatment‐related event (Table S4). Only two patients discontinued fedratinib due to treatment‐related anemia or thrombocytopenia (n = 1 each). No report of thrombocytopenia was associated with a major bleeding event.

For patients with a baseline platelet count of 50 to <100 × 10^9^/L (n = 33), the median number of fedratinib treatment cycles received was seven (range 1‐20), and for patients with platelet counts ≥100 × 10^9^/L (n = 64) was six (1‐18). Approximately 91% of patients with baseline platelet counts of 50 to <100 × 10^9^/L and 97% of patients with baseline platelet counts ≥100 × 10^9^/L received ≥80% of their intended fedratinib dose on‐study. Safety events were generally similar between the two baseline platelet count subgroups (Table S5), with the exception of a higher frequency of expected grade 3‐4 hematologic events in patients with lower baseline platelet counts: 46% and 49% of patients with platelet counts of 50 to <100 × 10^9^/L experienced grade 3‐4 anemia and thrombocytopenia, respectively, vs 34% and 8% of patients with baseline platelet counts ≥100 × 10^9^/L.

Patients aged ≤65 years (n = 41) received a median of seven treatment cycles (range 1‐20) and those aged >65 years (n = 56) received a median of six cycles (1‐18). Approximately 98% of patients aged ≤65 years and 93% of patients aged >65 years received ≥80% of their intended fedratinib dose. The incidences of TEAEs were generally similar between these age‐based subgroups.

No case of WE occurred in this study. Grade 3 encephalopathy was reported in one patient with underlying portal hypertension and esophageal varices, who experienced slight forgetfulness and no other neurological signs or symptoms. The investigator, external experts, and the Data Safety Monitoring Board for the study reached a consensus on a final diagnosis of hepatic encephalopathy and the patient experienced a full recovery.

## 4 DISCUSSION

In this population of heavily pretreated patients with poor prognostic features at baseline, approximately one‐third of all patients achieved the primary endpoint of ≥35% spleen volume reduction from baseline at EOC6, and most patients had reductions in spleen volume during fedratinib treatment. Patients in this study had substantial MF disease burden, as evidenced by large median spleen size and spleen volume at baseline, and almost all patients reported MFSAF symptoms at study entry. One‐third of patients had platelet counts below 100 × 10^9^/L and more than one‐half had hemoglobin levels below 10 g/dL. Clinically meaningful reductions in splenomegaly and symptom burden with fedratinib in the ITT Population are supported by analyses in patients who met stringent criteria for ruxolitinib relapsed/refractory or intolerant. Moreover, outcomes in the Sensitivity Analysis Cohort, in which patients were allowed sufficient exposure to fedratinib to determine lack or loss of response or intolerance, also strongly support findings in the ITT population.

Other JAK inhibitors tested in patients with MF previously treated with ruxolitinib are in late‐stage clinical development. In the phase III PERSIST2 trial of pacritinib, a JAK2/FLT3 inhibitor, 6 of 62 patients (10%) who had received prior ruxolitinib therapy achieved a ≥35% spleen volume reduction with pacritinib at 24 weeks.^30^ Similarly, in the phase III SIMPLIFY‐2 study of momelotinib, a JAK1/JAK2 inhibitor, 7 of 104 patients (7%) who had previously received ruxolitinib achieved a spleen volume response with momelotinib.^31^ These low response rates emphasize the difficulty of attaining future responses in patients with MF previously treated with ruxolitinib. Acknowledging differences in study designs and the absence of head‐to‐head comparisons, results of JAKARTA2 compare favorably with those for other JAK inhibitors in similar patient populations.

In the current study, more than 90% of patients with baseline platelet counts of 50 to <100 × 10^9^/L received ≥80% of their intended fedratinib dose; treatment was generally tolerable, and spleen volume and symptom response rates were comparable to rates for patients who entered the study with platelet counts ≥100 × 10^9^/L. Similarly, baseline platelet count did not significantly influence spleen response rates in the phase III JAKARTA trial of fedratinib in JAK‐inhibitor‐naive patients with intermediate‐2 or high‐risk MF.^32^ Even though ruxolitinib, the only other approved JAK inhibitor for MF, can be used at lower doses (5 or 10 mg twice‐daily) in patients with MF who have platelet counts of 50 to <100 × 10^9^/L, it may be at the expense of drug efficacy.^9^, ^33^

Hematologic events are anticipated with JAK inhibitors based on their mechanism of action.^34^ As expected, grade 3 or 4 cytopenias were more commonly reported in patients who began the study with platelet counts of 50 to <100 × 10^9^/L. Importantly, cytopenias were rarely cause for permanent fedratinib treatment discontinuation, suggesting that these events could be managed effectively with dose modifications, temporary treatment interruptions, and transfusions. No report of thrombocytopenia was associated with a major bleeding event.

The most frequent TEAEs in this study were low‐grade gastrointestinal events. Clinical data suggest that taking fedratinib with a high‐fat meal improves gastrointestinal tolerability with minimal effect on bioavailability.^35^ Strategies for prevention and management of gastrointestinal effects include prophylaxis for nausea or vomiting with antiemetics (eg, 5‐HT3 receptor antagonists; dimenhydrinate and anticholinergic and antimuscarinic agents can confound CNS symptoms and should be taken with caution), therapeutic use of antidiarrheals at onset of symptoms, and fedratinib dose modifications if toxicity continues despite supportive treatment.

As mentioned, fedratinib clinical trials were placed on clinical hold by the US FDA in November 2013 following reports of suspected WE, a neurologic emergency resulting from thiamine (vitamin B1) deficiency, and the clinical development program was subsequently terminated by the sponsor. The clinical hold was lifted in August 2017 after additional safety data were provided to the FDA. The fedratinib prescribing information includes a Boxed Warning for encephalopathy, including WE, based on eight reported cases observed in more than 600 patients treated with multiple doses of fedratinib in clinical trials.^21^ Among the eight suspected WE cases; seven patients were taking fedratinib 500 mg/day at the time of symptom onset. The one case that occurred with fedratinib 400 mg/day was a patient in JAKARTA2 who was determined in an independent review by external experts to have hepatic encephalopathy, not WE. While most events resolved (some involved persistent deficits, including memory loss, cognitive impairment, and dizziness), one patient with head and neck cancer metastatic to the brain and significant predisposing factors for WE, including difficulty eating and weight loss, had a fatal outcome. Retrospective analysis of the potential events suggested that all affected patients had considerable concomitant conditions known to predispose to WE in any population (eg, underlying malnutrition, nausea, vomiting, diarrhea).^36^, ^37^ Importantly, preclinical data from animal models show that fedratinib, administered at clinically‐relevant doses, does not inhibit thiamine transport either from the GI to plasma or from plasma to brain, nor does it lead to neurologic changes associated with thiamine deficiency.^38^, ^39^

Risk‐mitigation strategies for WE and gastrointestinal TEAEs, including routine monitoring of thiamine and thiamine supplementation as appropriate, and proactive treatment of gastrointestinal events with the use of anti‐emetics and antidiarrheals, are being evaluated in the ongoing fedratinib phase III clinical program (FREEDOM [ClinicalTrials.gov NCT03755518] and FREEDOM2 [NCT03952039]) assessing the efficacy and safety of fedratinib in patients with intermediate‐2 or high‐risk MF previously treated with ruxolitinib.

Early study termination of JAKARTA2 may have led to underestimation of fedratinib response. Spleen volume reductions of ≥35% from baseline occurred in a higher proportion of patients who had the opportunity to complete three cycles of fedratinib treatment than the rate reported in the ITT analysis at EOC6; thus, patients who were responding to fedratinib therapy may have been discontinued due to the clinical hold before a cycle six measure was taken and would have been considered nonresponders. Early termination also prevented assessment of longer‐term efficacy and safety of fedratinib treatment. Currently, the longest exposure to fedratinib therapy occurred in the extension portion of a phase I dose‐finding and expansion study of fedratinib in adult patients with MF (ClinicalTrials.gov NCT00631462, NCT00724334).^40^, ^41^ In an interim analysis from that study, 23 of 59 patients (39%) had received long‐term fedratinib treatment for a median 30 cycles (range 13‐44) at a median current fedratinib dose of 440 mg.^41^ No unexpected safety signals emerged during long‐term fedratinib therapy.^41^ Long‐term outcomes with fedratinib in patients previously treated with ruxolitinib are currently under investigation in the aforementioned FREEDOM and FREEDOM2 studies.

Eligibility criteria for the JAKARTA2 study required a relatively limited degree of ruxolitinib exposure as sufficient to determine ruxolitinib failure at enrollment. However, the median prior ruxolitinib treatment duration in the ITT Population was 10.7 months, and outcomes in the Stringent Criteria and Sensitivity Analysis cohorts, which included patients with greater prior ruxolitinib exposure than initially protocol‐specified, were consistent with those of the ITT Population.

This rigorous updated analysis of JAKARTA2 data demonstrates that patients with advanced MF who were substantially pretreated with ruxolitinib could attain robust spleen responses and reduced symptom burden with fedratinib. The efficacy of fedratinib was confirmed in the subgroup of JAKARTA2 patients who met stringent criteria for ruxolitinib relapsed, refractory, or intolerant, and in the sensitivity analysis comprising patients who were least affected by the fedratinib clinical hold and early study termination. Fedratinib is an important new treatment option for patients with MF, particularly those who have previously been treated with Ruxolitinib, as well as those patients with low pretreatment platelet counts or hemoglobin levels.

## Supporting information


**Appendix S1**. Supporting information.
