# Supplementary material for: Fedratinib in patients with myelofibrosis previously treated with ruxolitinib: An updated analysis of the JAKARTA2 study using stringent criteria for ruxolitinib failure
Source: Am J Hematol. 2020 Apr 17;95(6):594–603. doi: 10.1002/ajh.25777 (PMC7317815; doi:10.1002/ajh.25777)

**Supplementary Table 1. JAKARTA2 analysis populations**

| **ITT Population**  **(N=97)** | **Stringent Criteria Cohort**  **(n=79)** | **Sensitivity Analysis Cohort**  **(n=66)** |
| --- | --- | --- |
| Ruxolitinib resistant or intolerant per investigator discretion:  *Resistant*: Lack of response, evidence of disease progression, or loss of response to ruxolitinib following ≥14 days of treatment  *Intolerant*: Discontinuation due to unacceptable toxicity after any duration of ruxolitinib exposure | *Relapsed:* Ruxolitinib treatment for ≥3 months with spleen regrowth, defined as <10% SVR or <30% decrease in spleen size from baseline, following an initial response.* | Subgroup of patients within the Stringent Criteria Cohort who reached cycle 6 of fedratinib therapy or discontinued before cycle 6 for reasons other than “*study terminated by sponsor*” |
|  | *Refractory:* Ruxolitinib treatment for ≥3 months with <10% SVR or <30% decrease in spleen size from baseline. |  |
|  | *Intolerant*: Ruxolitinib treatment for ≥28 days complicated by development of RBC transfusion requirement (≥2 units per month for 2 months); or grade ≥3 thrombocytopenia, anemia, hematoma and/or hemorrhage while receiving ruxolitinib. |  |
| *Response to ruxolitinib is defined as a ≥35% reduction in spleen volume from baseline, or a ≥50% reduction in spleen size for baseline spleen sizes >10 cm below left costal margin (LCM); a non-palpable spleen for baseline spleen sizes between 5–10 cm below LCM; or not eligible for spleen response for baseline spleen <5 cm below LCM.  LCM, left costal margin; RBC, red blood cell; SVR, spleen volume reduction. | | |

**Supplementary Table 2. Changes in individual symptom scores with fedratinib at the end of cycle 6 for patients with Myelofibrosis Symptom Assessment Form (MFSAF) assessments at baseline and at the end of cycle 6**

|  | **MFSAF Analysis Population**  **(N=51)** | **Stringent Criteria Cohort**  **(N=44)** |
| --- | --- | --- |
|  | **Number of patients with TSS data at baseline and EOC6**  **Median percent change from baseline (range)** | |
| Night sweats | n=42  −76% (−100, +200) | n=36  −67% (−100, +200) |
| Pruritus | n=35  −44% (−100, +467) | n=31  −44% (−100, +467) |
| Abdominal discomfort | n=48  −46% (−100, +650) | n=42  −46% (−100, +650) |
| Early satiety | n=46  −51% (−100, +1800) | n=39  −51% (−100, +1800) |
| Pain under ribs on left side | n=41  −83% (−100, +600) | n=35  −83% (−100, +100) |
| Bone or muscle pain | n=45  −22% (−100, +1443) | n=38  −20% (−100, +1443) |
| EOC6, end of cycle 6; MFSAF, Myelofibrosis Symptom Assessment Form; TSS, total symptom score.  Patients with baseline score of 0 were not included in percent change in symptom score analyses (symptom change not evaluable). | | |

**Supplementary Table 3. Hematology and biochemistry laboratory abnormalities of interest**

|  | **ITT Population**  **(N=97)** | | **Stringent Criteria Cohort**  **(n=79)** | | **Sensitivity Analysis Cohort**  **(n=66)** | | |
| --- | --- | --- | --- | --- | --- | --- | --- |
|  | **Any Grade** | **Grade 3–4** | **Any Grade** | **Grade 3–4** | **Any Grade** | **Grade 3–4** |  |
| **Hematology,* n (%)** |  |  |  |  |  |  |  |
| Anemia | 96 (99) | 45 (46) | 79 (100) | 43 (54) | 66 (100) | 37 (56) |  |
| Thrombocytopenia | 68 (70) | 23 (24) | 58 (73) | 18 (23) | 51 (77) | 16 (24) |  |
| Neutrophil count decrease | 23 (24) | 7 (7) | 22 (28) | 7 (9) | 20 (30) | 7 (11) |  |
| **Biochemistry,* n (%)** |  |  |  |  |  |  |  |
| Creatinine increased | 72 (74) | 0 | 57 (72) | 0 | 48 (73) | 0 |  |
| AST increased | 46 (47) | 1 (1) | 39 (49) | 1 (1) | 33 (50) | 1 (2) |  |
| ALT increased | 44 (45) | 2 (2) | 41 (52) | 2 (3) | 35 (53) | 2 (3) |  |
| Lipase increased | 25 (26) | 8 (8) | 20 (25) | 6 (8) | 17 (26) | 5 (8) |  |
| Amylase increased | 17 (18) | 3 (3) | 15 (19) | 2 (3) | 10 (15) | 2 (3) |  |
| *Hematologic and biochemical events (worst grade, including baseline) were assessed by laboratory analysis.  ALT, alanine aminotransferase; AST, aspartate aminotransferase | | | | | | |  |

**Supplementary Table 4. Treatment-related TEAEs leading to permanent fedratinib discontinuation**

| **Preferred Term** | **Fedratinib 400 mg N = 97** | |
| --- | --- | --- |
|  | **Treatment-related TEAEs**  **n (%)** | |
|  | **Any Grade** | **Grade 3–4** |
| Patients with ≥ 1 treatment-related TEAE leading to permanent treatment discontinuation | 10 (10) | 8 (8) |
| Diarrhea | 2 (2) | 2 (2) |
| Nausea | 1 (1) | 0 |
| Vomiting | 1 (1) | 0 |
| Blood creatinine increased | 1 (1) | 0 |
| Gamma-glutamyltransferase increased | 1 (1) | 1 (1) |
| Platelet count decreased | 1 (1) | 1 (1) |
| Weight decreased | 1 (1) | 1 (1) |
| Thrombocytopenia | 1 (1) | 1 (1) |
| Anemia | 1 (1) | 1 (1) |
| Thrombotic thrombocytopenic purpura | 1 (1) | 1 (1) |
| Encephalopathy | 1 (1) | 1 (1) |

**Supplementary Table 5. Treatment-emergent adverse events (any grade) reported in ≥ 10% of all patients, by baseline platelet count***

|  | ITT Population (N=97) | |
| --- | --- | --- |
|  | Baseline Platelet Count | |
|  | 50 to <100 x 10^9^/L (n=33) | ≥100 x 10^9^/L (n=64) |
| Nausea | 20 (61) | 34 (53) |
| Diarrhea | 19 (58) | 41 (64) |
| Anemia | 19 (58) | 28 (44) |
| Thrombocytopenia | 18 (55) | 8 (13) |
| Vomiting | 16 (49) | 24 (38) |
| Constipation | 8 (24) | 12 (19) |
| Pruritus | 7 (21) | 10 (16) |
| Abdominal Pain | 6 (18) | 6 (9) |
| Epistaxis | 6 (18) | 2 (3) |
| Dyspnea | 6 (18) | 6 (9) |
| Pyrexia | 5 (15) | 6 (9) |
| Bone pain | 5 (15) | 2 (3) |
| Urinary tract infection | 4 (12) | 8 (13) |
| Pain in extremity | 4 (12) | 2 (3) |
| Decreased appetite | 4 (12) | 5 (8) |
| Transfusion dependence | 4 (12) | 4 (6) |
| Fatigue | 3 (9) | 12 (19) |
| Headache | 3 (9) | 10 (16) |
| Dizziness | 3 (9) | 8 (13) |
| Asthenia | 2 (6) | 9 (14) |
| Weight decreased | 1 (3) | 8 (13) |
| Cough | 0 | 13 (20) |
| *Sorted by decreasing frequency in the subgroup of patients with platelet counts of 50 to <100 × 10^9^/L at baseline.  MedDRA version 20.1. | | |

**Supplementary Figure 1. JAKARTA2 patient populations and reasons for ruxolitinib discontinuation**

**
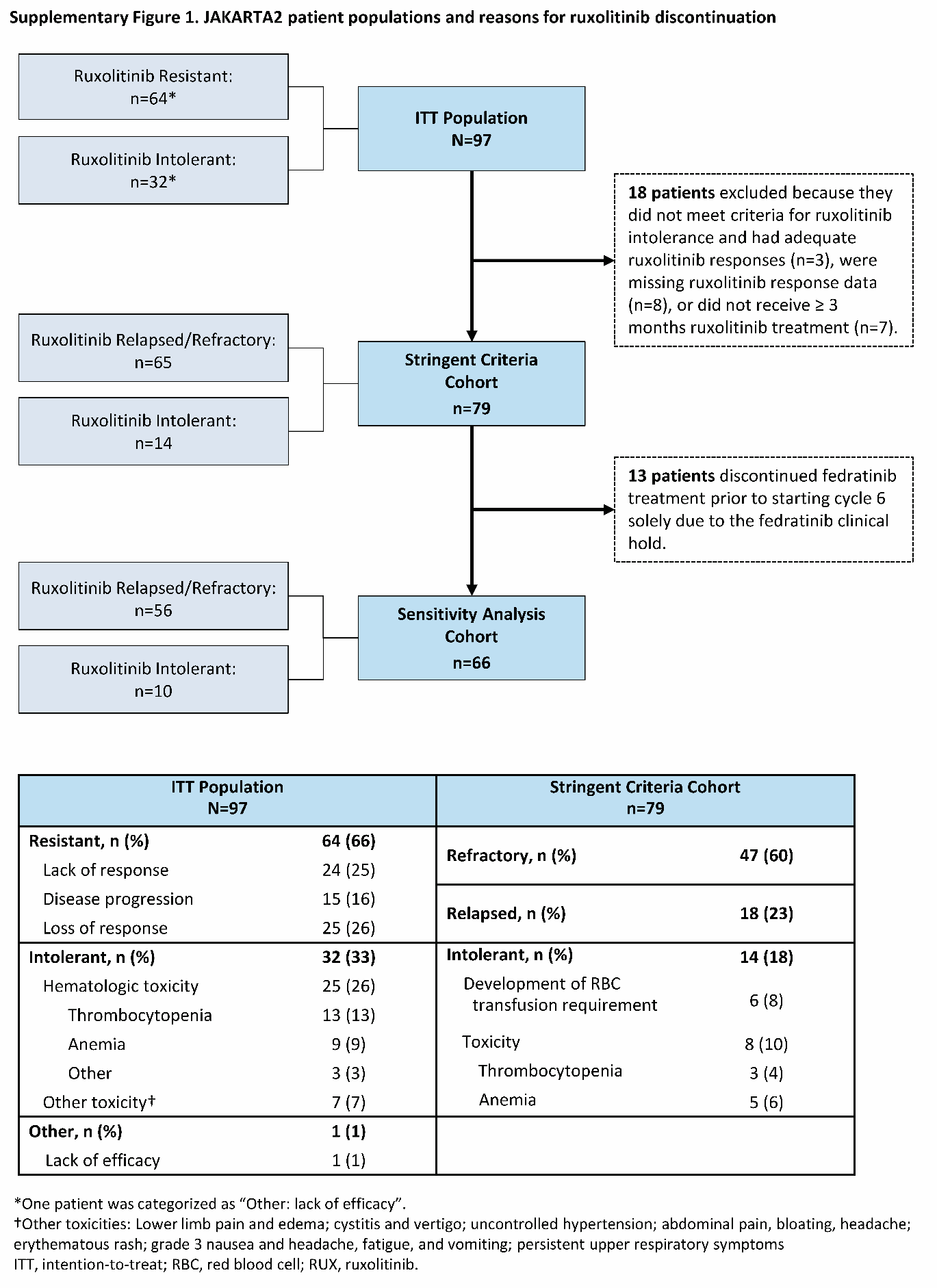
**

**Supplementary Figure 2. Kaplan-Meier estimated duration of spleen volume response (ITT Population)**


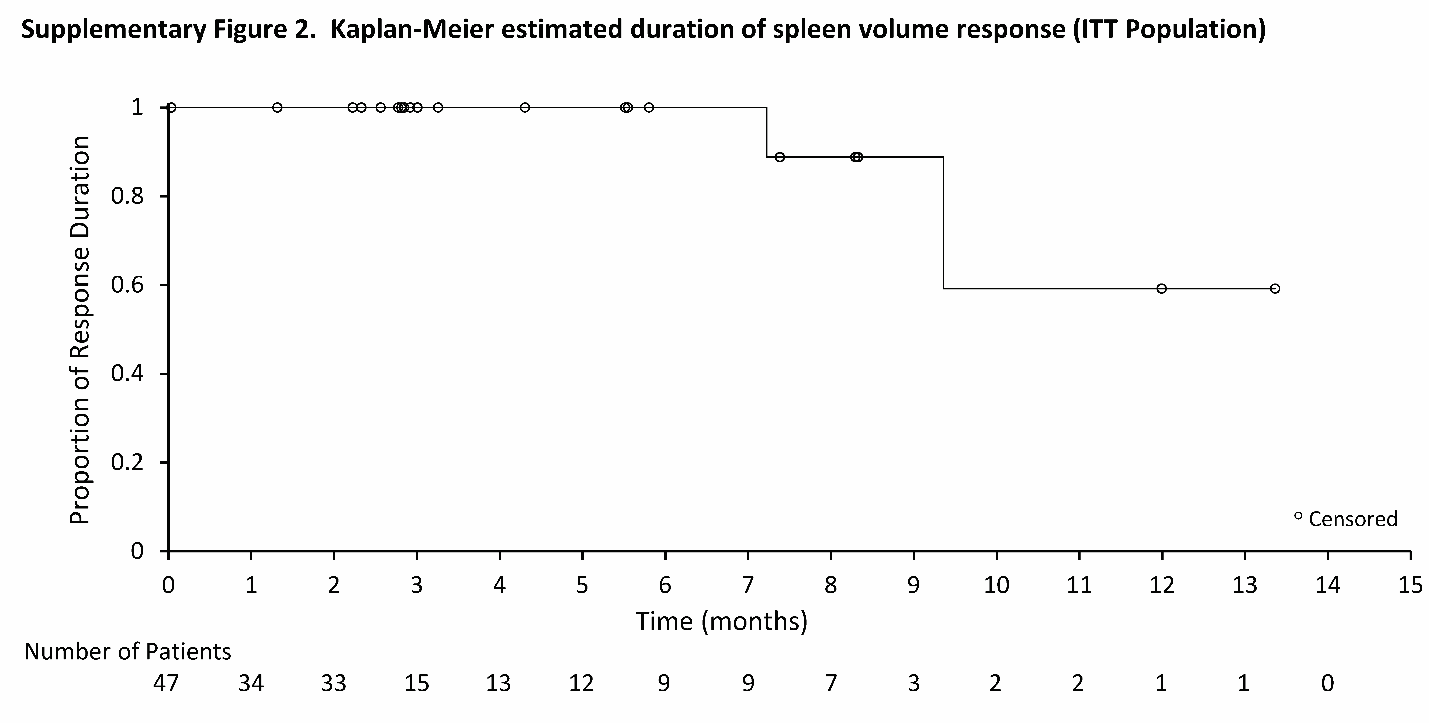

Supplement: Supplementary file 1 — Appendix S1. Supporting information. [file AJH-95-594-s001.docx]
